# Oleaginicity of the yeast strain *Saccharomyces cerevisiae* D5A

**DOI:** 10.1186/s13068-018-1256-z

**Published:** 2018-09-24

**Authors:** Qiaoning He, Yongfu Yang, Shihui Yang, Bryon S. Donohoe, Stefanie Van Wychen, Min Zhang, Michael E. Himmel, Eric P. Knoshaug

**Affiliations:** 1 State Key Laboratory of Biocatalysis and Enzyme Engineering, Hubei Collaborative Innovation Center for Green Transformation of Bio-resources, Environmental Microbial Technology Center of Hubei Province, Hubei Key Laboratory of Industrial Biotechnology, College of Life Sciences, Hubei University, Wuhan, 430062 China; 2 National Bioenergy Center, National Renewable Energy Laboratory, Golden, 80401 USA; 3 Biosciences Center, National Renewable Energy Laboratory, Golden, 80401 USA

**Keywords:** Oleaginous yeast, *Saccharomyces cerevisiae*, Transcriptomics, RNA-Seq, Lipid accumulation, Triacylglycerol (TAG), Nitrogen assimilation

## Abstract

**Background:**

The model yeast*, Saccharomyces cerevisiae*, is not known to be oleaginous. However, an industrial wild-type strain, D5A, was shown to accumulate over 20% storage lipids from glucose when growth is nitrogen-limited compared to no more than 7% lipid accumulation without nitrogen stress.

**Methods and results:**

To elucidate the mechanisms of *S. cerevisiae* D5A oleaginicity, we compared physiological and metabolic changes; as well as the transcriptional profiles of the oleaginous industrial strain, D5A, and a non-oleaginous laboratory strain, BY4741, under normal and nitrogen-limited conditions using analytic techniques and next-generation sequencing-based RNA-Seq transcriptomics. Transcriptional levels for genes associated with fatty acid biosynthesis, nitrogen metabolism, amino acid catabolism, as well as the pentose phosphate pathway and ethanol oxidation in central carbon (C) metabolism, were up-regulated in D5A during nitrogen deprivation. Despite increased carbon flux to lipids, most gene-encoding enzymes involved in triacylglycerol (TAG) assembly were expressed at similar levels regardless of the varying nitrogen concentrations in the growth media and strain backgrounds. Phospholipid turnover also contributed to TAG accumulation through increased precursor production with the down-regulation of subsequent phospholipid synthesis steps. Our results also demonstrated that nitrogen assimilation via the glutamate–glutamine pathway and amino acid metabolism, as well as the fluxes of carbon and reductants from central C metabolism, are integral to the general oleaginicity of D5A, which resulted in the enhanced lipid storage during nitrogen deprivation.

**Conclusion:**

This work demonstrated the disequilibrium and rebalance of carbon and nitrogen contribution to the accumulation of lipids in the oleaginous yeast *S. cerevisiae* D5A. Rather than TAG assembly from acyl groups, the major switches for the enhanced lipid accumulation of D5A (i.e., fatty acid biosynthesis) are the increases of cytosolic pools of acetyl-CoA and NADPH, as well as alternative nitrogen assimilation.

**Electronic supplementary material:**

The online version of this article (10.1186/s13068-018-1256-z) contains supplementary material, which is available to authorized users.

## Background

Lipid-based biofuels produced from microorganisms are attractive substitutes for fossil fuels due to their sustainable and secure supply [1]. These microorganisms can be grown on a variety of lignocellulosic carbon sources negating the need for large agricultural areas of dedicated oil crops [2]. However, aerobic fermentation is expensive, compounds in lignocellulosic hydrolysates can inhibit fermentation, extraction of the oil can be energy intensive from dry biomass or inefficient from wet biomass, and if not carefully monitored, the yeast will begin to utilize their own lipid stores reducing productivity [2–6]. As current lipid productivities are not sufficient for producing lipid-based biofuels economically, a significant research to increase lipid accumulation or include the production of co-products to enable lipid-based biofuel production is ongoing [2, 3, 7]. Lipid accumulation in yeast can be through de novo biosynthesis, the fermentation of hydrophilic substrates such as sugars or other carbohydrates to lipids, or through ex novo biosynthesis, the conversion of hydrophobic substrates such as oil and alkanes [6, 8]. Studies of oleaginous microorganisms, such as microalgae, yeasts, filamentous fungi, and bacteria, have revealed that de novo lipid metabolism is dependent on environmental conditions, particularly nutrient shortages with an excess of carbohydrates [1, 9–11], while ex novo biosynthesis is not [8]. Among the potential limiting nutrients, nitrogen limitation has emerged as a reliable and efficient strategy to promote lipid production.

Lipid biosynthesis is a complex process involving many metabolic reactions in pathways spanning multiple cellular organelles in eukaryotic cells. Fatty acids (FAs) mainly serve as intermediates in lipid biosynthesis. Triacylglycerols (TAGs) are formed from glycerol and FAs, and are primarily found in lipid bodies for storage [12]. During de novo fatty acid biosynthesis, acetyl-CoA is carboxylated to malonyl-CoA by acetyl-CoA carboxylase (ACC1), which is a well-known limiting step [12–14]. With the help of the fatty acid synthase (FAS) complex, palmitoyl-CoA and stearoyl-CoA are produced and shuttled into the endoplasmic reticulum (ER) for TAG biosynthesis [15]. Substantial effort has been made to produce biofuels from sugars by over-expressing three primary genes involved in fatty acid biosynthesis (*ACC1*, *FAS1*, and *FAS2*); as well as knocking-out fatty-acyl-CoA synthetase 1 and 4 (*FAA1* and *FAA4*) [16, 17]. The catabolism of long-chain fatty acids starts from their activation by FAA1 and FAA4 in the cytoplasm followed by transport into peroxisomes, whereas medium-chain fatty acids are directly transported to and then activated by FAA2 in peroxisomes [14]. Moreover, the over-expression of fatty-acyl-CoA synthetase-encoding gene FAA3 and diacylglycerol acyl-transferase DGA1 increases lipid accumulation, especially storage lipids or TAG [18].

*Saccharomyces cerevisiae* is a well-studied model eukaryotic microorganism, and broadly used for commercial-scale ethanol production due to its growth robustness, capacity for high-density fermentations, and resistance to phage contamination [19–23]. *S. cerevisiae* is also well known for robust alcoholic fermentation of various pretreated lignocellulosic feedstocks for renewable bioethanol production. The *S. cerevisiae* strain, D5A, has previously been used to ferment pretreated switchgrass, rice straw, distiller’s grains, lodgepole pine, and microalgal feedstocks [24–26]. D5A was found to tolerate inhibitory hydrolysate compounds present in pretreated hardwoods [27], as well as 1% (v/v) butanol [28].

The classical definition of an oleaginous yeast is the one that accumulates greater than 20% dry cell weight (dcw) as lipids. *S. cerevisiae* is not known as being oleaginous and typically only accumulates 10–15% of its dcw as lipids [7, 9, 17, 28–30]. Oleaginous yeasts, such as *Rhodosporidium toruloides*, *Lipomyces starkeyi*, and *Yarrowia lipolytica*, can typically accumulate 25% to greater than 60% lipids [15, 31]. The previous engineering efforts in *S. cerevisiae* showed a promising increase in lipid content by over-expressing a type 1 plant *DGA* [29] or by deleting the global regulator, AMP-activated protein kinase SNF1, which has previously been shown to impact the control of energy metabolism, carbon source utilization, and lipid accumulation in *S. cerevisiae* [32]. Although the total lipid production of *S. cerevisiae* from glycerol was less than 12% dcw, it was nearly doubled over that of wild-type control [32] and up to 17% dcw lipids were accumulated by over-expressing *FAS1*, *FAS2*, and *ACC1*. These conditions also led to the production of low titers of ethanol (up to 4 g/L) [17].

Systems biology is an excellent tool for probing regulatory mechanisms and has been demonstrated extensively for *S. cerevisiae* [23, 33, 34]. With the advance of next-generation sequencing (NGS) technology, RNA-Seq-based transcriptomics made it possible to reveal the novel aspects of the genetic and molecular mechanisms of stress responses in oleaginous yeasts [35]. However, only a few studies have focused on the transcriptomics of lipid accumulation [33, 36]. In this study, the lipid accumulation mechanism of a wild-type industrial strain of *S. cerevisiae* D5A was investigated and compared to the non-oleaginous haploid laboratory strain, BY4741, using RNA-Seq transcriptomics under nitrogen-repleted or -deprived conditions. We elucidate the biological mechanisms critical for carbon allocation and metabolism enabling yeast oleaginicity.

## Results and discussion

### Lipid and metabolite contents differed markedly between *S. cerevisiae* D5A and BY4741

**Fig. 1:**
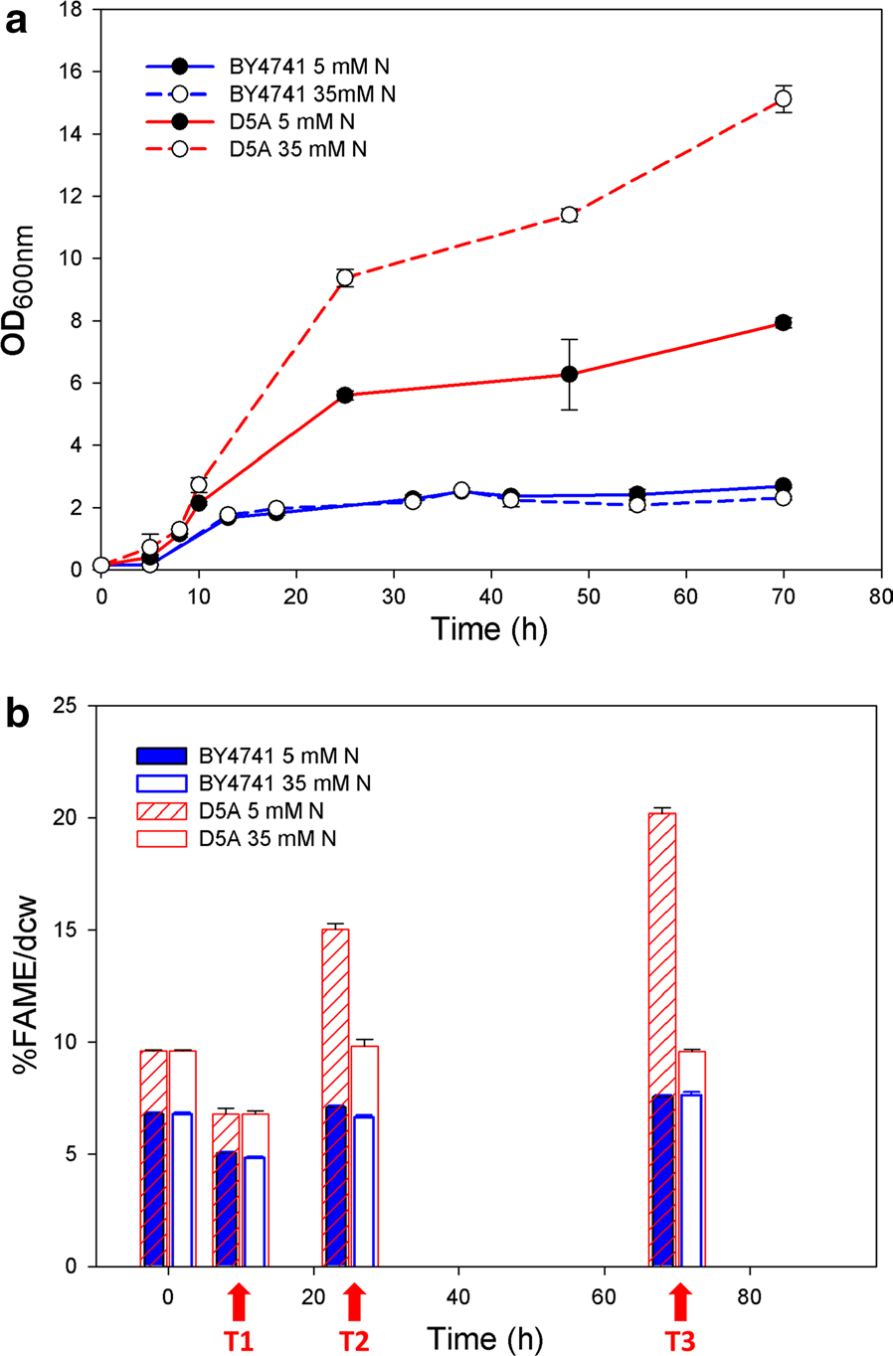
Growth (**a**) and FAME content (**b**) in strains D5A and BY4741. Red arrows indicate samples used for transcriptomics as they clearly show the induction of lipid production from T1 to T2 and T3 in D5A. Data shown as the mean ± standard deviation of duplicate OD_600_ samples and triplicate FAME samples

We previously discovered that *S. cerevisiae* strain D5A is naturally oleaginous compared to other industrially relevant strains [37]. Thus, a transcriptomic study to determine the underlying mechanisms in D5A compared to the laboratory haploid strain of BY4741 was carried out in this work. Growth and lipid accumulation of D5A and BY4741 with 5 mM (nitrogen deprivation, − N) or 35 mM (NH_4_)_2_SO_4_ (nitrogen replete, + N) were determined. The growth of D5A was better than that of BY4741 in both conditions (Fig. 1). No significant differences in growth or lipid content were found for BY4741 between N-deprived (OD_600nm_ = 2.7) and N-repleted (OD_600nm_ = 2.3) conditions. However, the culture density of D5A under N replete (OD_600nm_ = 15.1) was significantly higher than that in N-deprived (OD_600nm_ = 7.9) conditions (Fig. 1a). Clearly, cell proliferation decreased during N deprivation in D5A when compared to that under N-repleted condition, which is consistent with other oleaginous yeasts and microalgae species, reflecting the decrease of growth rate upon N limitation [10, 34, 38, 39].

During the initial growth phase (**T1**), the fatty acid methyl ester (FAME) content in both strains decreased from that of the seed cultures in both N concentrations (Fig. 1b). As FAME accumulation began (**T2**), FAME content increased markedly in D5A to 15.0% and 9.8% dcw in N-deprived and -repleted conditions, respectively. In contrast, FAME content in BY4741 only increased to 7.1% and 6.7% dcw, respectively. In the late oleaginous phase (**T3**), FAME content in D5A continued to rise to 20.2% dcw in the N-deprived condition with no further increase in the N-repleted condition. Conversely, FAME content did not increase above 8% in BY4741 (Fig. 1b).

Lipids from *S. cerevisiae* D5A consist primarily of four species: the saturated fatty acids palmitic (C16:0) and stearic (C18:0) and the monounsaturated fatty acids palmitoleic (C16:1n7) and oleic (C18:1n9) (Tables 1, 2) [40, 41]. The fatty acid profile of D5A differs from that of typical oleaginous yeasts. Whereas, in D5A, the fatty acids C16:0, C16:1n7, and C18:1n9 make up > 90% of the fatty acids, in general, in other oleaginous yeasts, e.g., *Rhodotorula*, *Rhodosporidium*, *Yarrowia, Trichosporon*, and *Lipomyces* species, the fatty acids C16:0 and C18:1n9 make up the bulk of the fatty acids with minor contributions from C16:1n7, C18:0, and C18:2n6 [5, 6, 8, 11, 39]. In general, the change in fatty acid composition of these two strains differed as the culture cells matured. In D5A, the overall trend, irrespective of N stress, was toward an increased amount of monounsaturated lipids C16:1n7 and C18:1n9, whereas their cognate-saturated fatty acids decreased. In contrast, BY4741 showed the opposite response having an increase in saturated fatty acid species, while their cognate monounsaturated species decreased, also irrespective of N stress.

**Table 1:** FAME composition in D5A during different N conditions and oleaginous phases

	*S. cerevisiae* D5A					
	5 mM (− N)			35 mM (+ N)		
	T1	T2	T3	T1	T2	T3
C10:0	0.27 ± 0.03	0.05 ± 0.02	0.06 ± 0.01	0.39 ± 0.04	0.04 ± 0.05	0 ± 0
C12:0	1.03 ± 0.02	0.41 ± 0.00	0.39 ± 0.03	0.94 ± 0.07	0.18 ± 0.02	0.22 ± 0.06
C14:0	2.16 ± 0.03	1.33 ± 0.02	1.12 ± 0.03	2.11 ± 0.22	0.97 ± 0.07	0.82 ± 0.11
C14:1	0.46 ± 0.03	0.55 ± 0.02	0.53 ± 0.03	0.38 ± 0.06	0.3 ± 0.03	0.52 ± 0.03
C16:0	15.44 ± 0.35	10.79 ± 0.16	10.58 ± 0.19	16.49 ± 0.15	10.86 ± 0.31	8.63 ± 0.59
C16:1n7	41.85 ± 0.23	42.4 ± 0.40	46.77 ± 0.52	38.76 ± 0.94	38.51 ± 0.81	45.03 ± 1.10
C18:0	3.55 ± 0.08	4.23 ± 0.09	4.11 ± 0.14	4.54 ± 0.35	4.9 ± 0.24	3.77 ± 0.03
C18:1n7	0.55 ± 0.05	0.75 ± 0.02	1.08 ± 0.03	0.5 ± 0.06	1.78 ± 0.02	1.81 ± 0.05
C18:1n9	34.69 ± 0.18	39.48 ± 0.52	35.32 ± 0.32	35.88 ± 0.75	42.46 ± 0.45	39.17 ± 0.34

Data shown as the mean of triplicate samples with standard deviation

**Table 2:** FAME composition in BY4741 during different N conditions and oleaginous phases

	*S. cerevisiae* BY4741					
	5 mM (− N)			35 mM (+ N)		
	T1	T2	T3	T1	T2	T3
C10:0	0.81 ± 0.01	0.77 ± 0.00	0.69 ± 0.02	0.94 ± 0.10	0.85 ± 0.03	0.88 ± 0.00
C12:0	1.97 ± 0.05	1.82 ± 0.02	1.68 ± 0.01	2.01 ± 0.09	1.75 ± 0.04	1.71 ± 0.03
C14:0	1.86 ± 0.02	1.16 ± 0.01	1.03 ± 0.05	1.91 ± 0.15	1.15 ± 0.08	1.12 ± 0.05
C14:1	0.1 ± 0.03	0.05 ± 0.01	0.05 ± 0.01	0.08 ± 0.02	0.05 ± 0.04	0.09 ± 0.02
C16:0	23.92 ± 0.13	28.44 ± 0.15	28.78 ± 0.28	24.23 ± 0.24	24.49 ± 0.33	24.44 ± 0.12
C16:1n7	41.12 ± 0.35	35.92 ± 0.22	35.1 ± 0.31	40.81 ± 0.31	39.79 ± 0.20	39.36 ± 0.28
C18:0	4.76 ± 0.04	7.11 ± 0.05	8.27 ± 0.14	4.86 ± 0.14	5.46 ± 0.19	5.84 ± 0.18
C18:1n7	1.14 ± 0.11	2.04 ± 0.06	2.13 ± 0.08	1.01 ± 0.16	2.46 ± 0.28	2.66 ± 0.26
C18:1n9	24.29 ± 0.10	22.43 ± 0.04	22.15 ± 0.01	24.14 ± 0.60	23.94 ± 0.24	23.75 ± 0.22

Data shown as the mean of triplicate samples with standard deviation

D5A consumed glucose more rapidly compared to BY4741 in both conditions (Additional file 1: Figure S1A). Glucose was depleted within 21 h in N-repleted condition in D5A, accompanied with maximum ethanol production (Additional file 1: Figure S1B). Similarly, glycerol peaked within 21 h, while acetic acid peaked within 30 h. Glucose consumption in the N-deprived condition took considerably longer with 95% being consumed within 72 h, while ethanol and acetic acid peaked and then declined after approximately 30 h. Glycerol increased gradually reaching a final titer of 4 g/L. In comparison, only approximately 55% of the glucose was utilized by BY4741 within 56 h in both N conditions. All metabolite concentrations gradually increased in both N conditions (Additional file 1: Figure S1C).

### Transcriptional profiles are different between D5A and BY4741 during N deprivation

Global transcriptional responses were examined by NGS-based RNA-Seq. Three growth and lipid accumulation time points (**T1**, **T2**, and **T3**) with biological replicate data were used for statistical analysis to understand transcriptomic profiling under N-deprived and N-repleted conditions in D5A and BY4741. Sample quality and correlations between the duplicate samples are high (Additional file 2: Table S1). Transcript levels in D5A and BY4741 under N-deprived and N-replete conditions at the different time points were quantified by the log_2_-transformed Reads Per Kilobase per Million mapped reads (RPKM) values (Additional file 3: Table S2).

Differential gene expression levels for each time point in N-depleted conditions and strain comparison for both N-depleted and -repleted conditions are included in Additional file 4: Table S3. These differentially expressed genes were further subdivided into several pathways important to lipid production (Additional file 5: Table S4A–H). From these differentially expressed genes, those that were significantly different for D5A during N deprivation or compared to the non-oleaginous BY4741 during N deprivation were identified and grouped according to metabolic pathways for FA and TAG metabolism, nitrogen metabolism, amino acid metabolism, Embden–Meyerhof–Parnas (EMP) pathway, pentose phosphate pathway (PPP), and tricarboxylic acid (TCA) cycle (Table 3). In general, genes involved in FA biosynthesis, N metabolism, and amino acid metabolism were up-regulated in D5A during N deprivation. In addition, genes involved in the TCA cycle, glycolysis, and the glyoxylate cycle were increased in D5A compared to BY4741.

**Table 3:** Subset of genes with significant differential transcript levels in D5A during nitrogen-deprived condition (5 mM, − N) compared to nitrogen-repleted condition (35 mM, + N) in time points of T1, T2, and T3, as well as D5A compared to BY4741

Name	D5A (− N/+ N) (Log_2_)	D5A/BY4741 (Log_2_)	Function
*FA/TAG synthesis*			
OLE1	1.2	4.5	Delta(9) fatty acid desaturase
ELO3	1.5	0.2	Elongase
PIS1	1.1	− 0.3	Phosphatidylinositol synthase
POX1	− 1.4	2.4	Fatty-acyl coenzyme A oxidase
FAA1	− 1.4	0.8	Long-chain fatty-acyl-CoA synthetase
*N metabolism*			
GLT1	1.6	1.0	NAD(+)-dependent glutamate synthase (GOGAT)
GLN1	1.9	0.00	Glutamine synthetase (GS)
GDH1	1.4	− 0.3	NADP(+)-dependent glutamate dehydrogenase
GDH2	0.6	0.5	NAD(+)-dependent glutamate dehydrogenase
*AA metabolism*			
ARO8	3.4	− 1.5	Aromatic aminotransferase I
LEU2	2.4	6.9	Beta-isopropylmalate dehydrogenase (IMDH)
CHA1	2.7	− 3.6	Catabolic l-serine (l-threonine) deaminase
BAT1	3.3	− 2.5	Mitochondrial branched-chain amino acid (BCAA) aminotransferase
ASN1	2.4	− 3.1	Asparagine synthetase
*EMP*			
HXK2	2.9	− 0.3	Hexokinase isoenzyme 2
TDH1	− 3.8	1.4	Glyceraldehyde-3-phosphate dehydrogenase (GAPDH)
HXK1	− 3.2	1.4	Hexokinase isoenzyme 1
GLK1	− 3.0	1.9	Glucokinase
GPM1	− 2.1	− 0.3	Tetrameric phosphoglycerate mutase
FBP1	− 2.1	1.6	Fructose-1,6-bisphosphatase
PYK2	− 2.1	− 0.6	Pyruvate kinase
PGK1	− 1.9	− 0.1	3-Phosphoglycerate kinase
*PPP*			
TKL1	2.4	− 0.1	Transketolase
SOL3	1.4	− 326	6-Phosphogluconolactonase
GND1	1.1	0.3	6-Phosphogluconate dehydrogenase (decarboxylating)
TKL2	− 3.3	3.0	Transketolase
TAL1	− 2.1	0.3	Transaldolase
ZWF1	− 1.7	− 1.0	Glucose-6-phosphate dehydrogenase (G6PD)
*TCA cycle*			
PYC1	− 2.0	0.3	Pyruvate carboxylase isoform
CIT1	− 3.3	1.1	Citrate synthase
CIT3	− 2.6	− 0.3	Dual specificity mitochondrial citrate and methylcitrate synthase
ACO1	− 1.2	2.5	Aconitase
SDH1	− 1.5	1.8	Flavoprotein subunit of succinate dehydrogenase
MDH1	− 2.3	0.8	Mitochondrial malate dehydrogenase
SDH2	− 1.6	1.9	Iron–sulfur protein subunit of succinate dehydrogenase
FUM1	− 1.0	1.3	Fumarase
KGD1	− 1.5	1.0	mitochondrial *α*-ketoglutarate dehydrogenase complex subunit
KGD2	− 1.1	1.7	Dihydrolipoyl transsuccinylase
ICL1	− 1.4	1.4	Isocitrate lyase
*Other*			
YAT1	− 4.1	0.5	Outer mitochondrial carnitine acetyltransferase
CIT2	− 4.9	− 0.4	Citrate synthase
ICL1	− 1.4	1.4	Isocitrate lyase
ACS1	− 3.5	2.1	Acetyl-coA synthetase isoform
PDC6	1.5	0.7	Minor isoform of pyruvate decarboxylase
ADH4	− 3.3	4.0	Alcohol dehydrogenase isoenzyme type IV
ALD6	− 1.2	1.3	Cytosolic aldehyde dehydrogenase
GPP1	1.2	− 0.5	Constitutively expressed dl-Glycerol-3-phosphate phosphatase
GPP2	1.5	− 0.7	dl-Glycerol-3-phosphate phosphatase involved in glycerol biosynthesis

All transcriptional differences are shown as the mean of duplicate log_2_-based values

### Transcripts involved in fatty acid biosynthesis are up-regulated in D5A during nitrogen deprivation

**Fig. 2:**
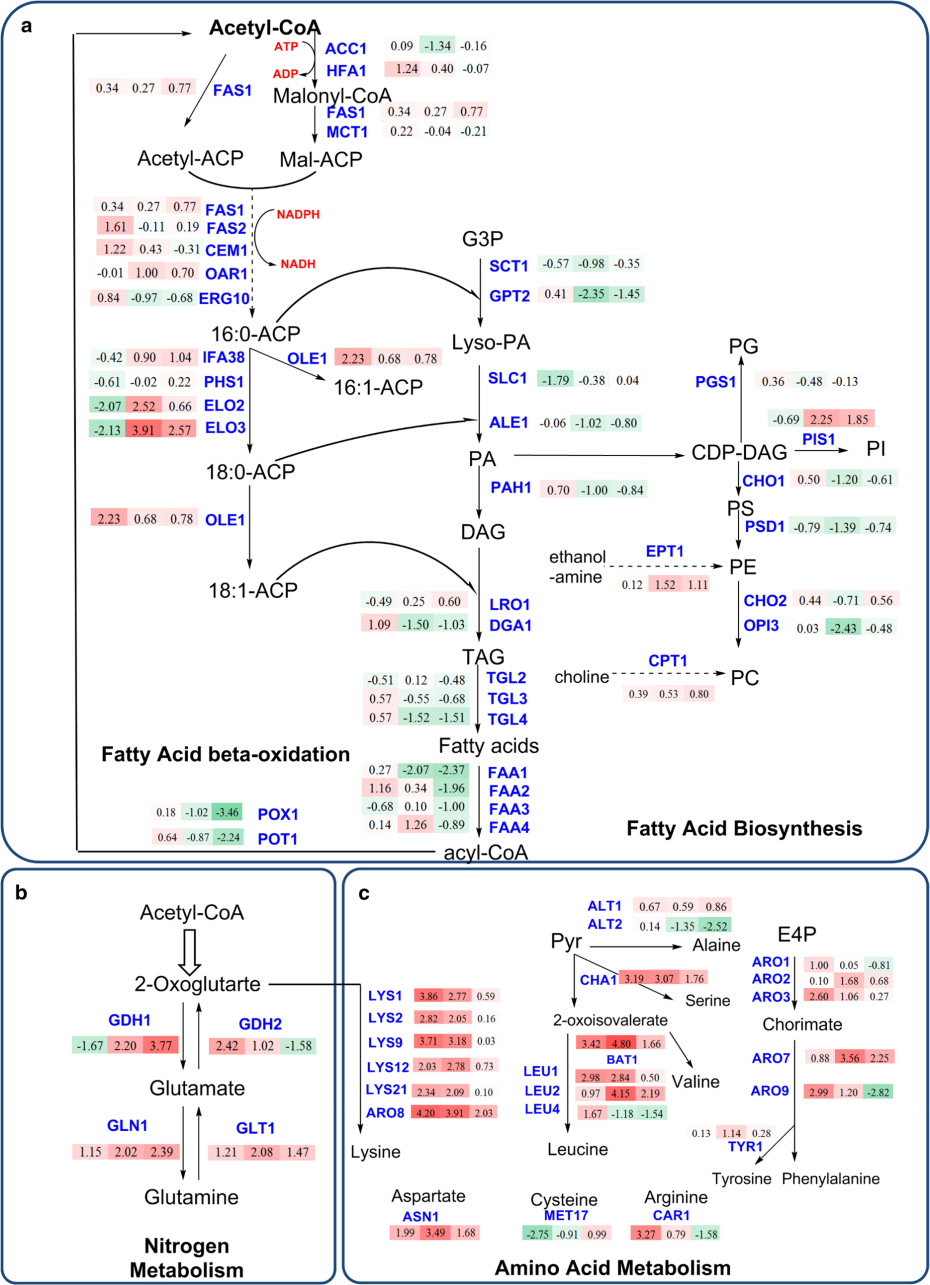
Transcript levels of genes in metabolic pathways of fatty acid biosynthesis (**a**), nitrogen metabolism (**b**), and amino acid metabolism (**c**) in D5A during N deprivation [5 mM (NH_4_)_2_SO_4_, − N] compared to N replete [35 mM (NH_4_)_2_SO_4_, + N]. The numbers in the shaded rectangles next to blue gene names indicate the log_2_-transformed fold changes in time points T1, T2, and T3, from left to right, respectively. Shades of green indicate the respective degree of which the gene is down-regulated and shades of red indicate the respective degree of which the gene is up-regulated. All transcriptional differences are shown as the mean of duplicate log_2_-based values

**Fig. 3:**
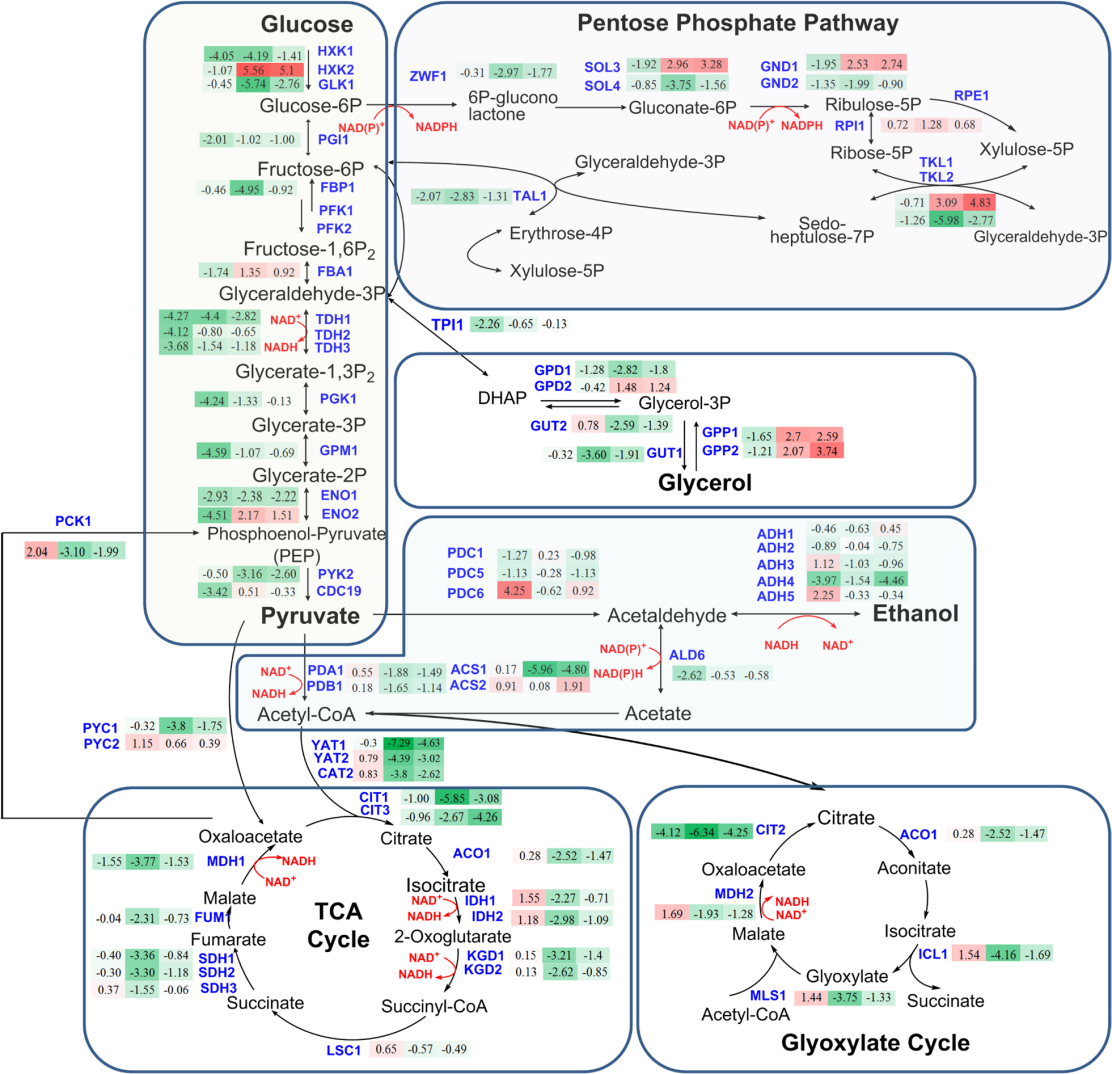
Transcript levels of genes in central carbon metabolism in D5A during N deprivation [5 mM (NH_4_)_2_SO_4_, − N] compared to N replete [35 mM (NH_4_)_2_SO_4_, + N]. The numbers in the shaded rectangles next to blue gene names indicate the log_2_-transformed fold changes in time points T1, T2, and T3, from left to right, respectively. Shades of green indicate the respective degree of which the gene is down-regulated and shades of red indicate the respective degree of which the gene is up-regulated. All transcriptional differences are shown as the mean of duplicate log_2_-based values

Due to the observed increase in lipid content in D5A as nitrogen became limiting, transcript levels were analyzed for lipid (fatty acid, triacylglycerol, and phospholipid) biosynthesis, nitrogen and amino acid metabolism, and central carbon metabolism pathways (Figs. 2, 3). During N deprivation, the increase in the carbon/nitrogen ratio (C/N) led to the accumulation of lipid in D5A, especially by the stationary phase (time point **T3**), which is consistent with other oleaginous yeasts [36, 42] and microalgae [43, 44], whereupon excess C is reallocated and diverted to metabolic pathways for the production of storage lipids [45]. The formation of lipid molecules begins when acyl-residues are transferred from an acyl-carrier protein (ACP) by an intrinsic acyl-transferase to coenzyme A (CoA), yielding long-chain acyl-CoAs by cytosolic fatty acid synthase (FAS). In this initial step, acetyl-CoA is carboxylated through the addition of CO_2_ to malonyl-CoA by acetyl-CoA carboxylase (ACC, encoded by *ACC1* and *HFA1*) [16]. Subsequently, malonyl-CoA is formed by multifunctional FAS that consists of two subunits encoded by *FAS1* and *FAS2*. During N deprivation in D5A, the transcript level of *FAS*, *HFA1*, and *CEM1* (fatty acid synthase) was increased significantly in time point of **T1** (Fig. 2), indicating increased flux of acyl-CoA towards lipid biosynthesis. Over-expression of *ACC1* combined with certain FAS and diacylglycerol acyl-transferase (DGA) genes resulted in increased lipid content [17]. However, due to the inhibition of SNF1 on the ACC1 activity by phosphorylation, over-expression of *ACC1* only in *S. cerevisiae* has a little effect on FA biosynthesis [46]. In this study, there were no significant changes in transcript abundance observed for *SNF1,* suggesting that the role of intact SNF1 in lipid biogenesis is more than simply transcript abundance. However, clearly, the complete absence of the SNF1 protein does promote lipid accumulation [37, 47] and influences the expression levels of genes involved in lipid accumulation in response to N deprivation [32]. In addition, the regulation of post-translational modification, such as the phosphorylation of ACC1 by SNF1, may also play a role in lipid accumulation.

After the initial formation of long-chain acyl-CoAs, elongases (ELO) act to lengthen the fatty acid chains. During N deprivation, the transcriptional level of *ELO3* increased dramatically in **T2** (15.0-fold) and **T3** (5.9-fold) (Fig. 2). In addition, fatty acid desaturase (OLE1), governing the removal of H from C16:0 and C18:0 to form monounsaturated fatty acids C16:1 and C18:1 [48, 49], was also up-regulated for 2.4-fold in D5A (Table 3).

**Fig. 4:**
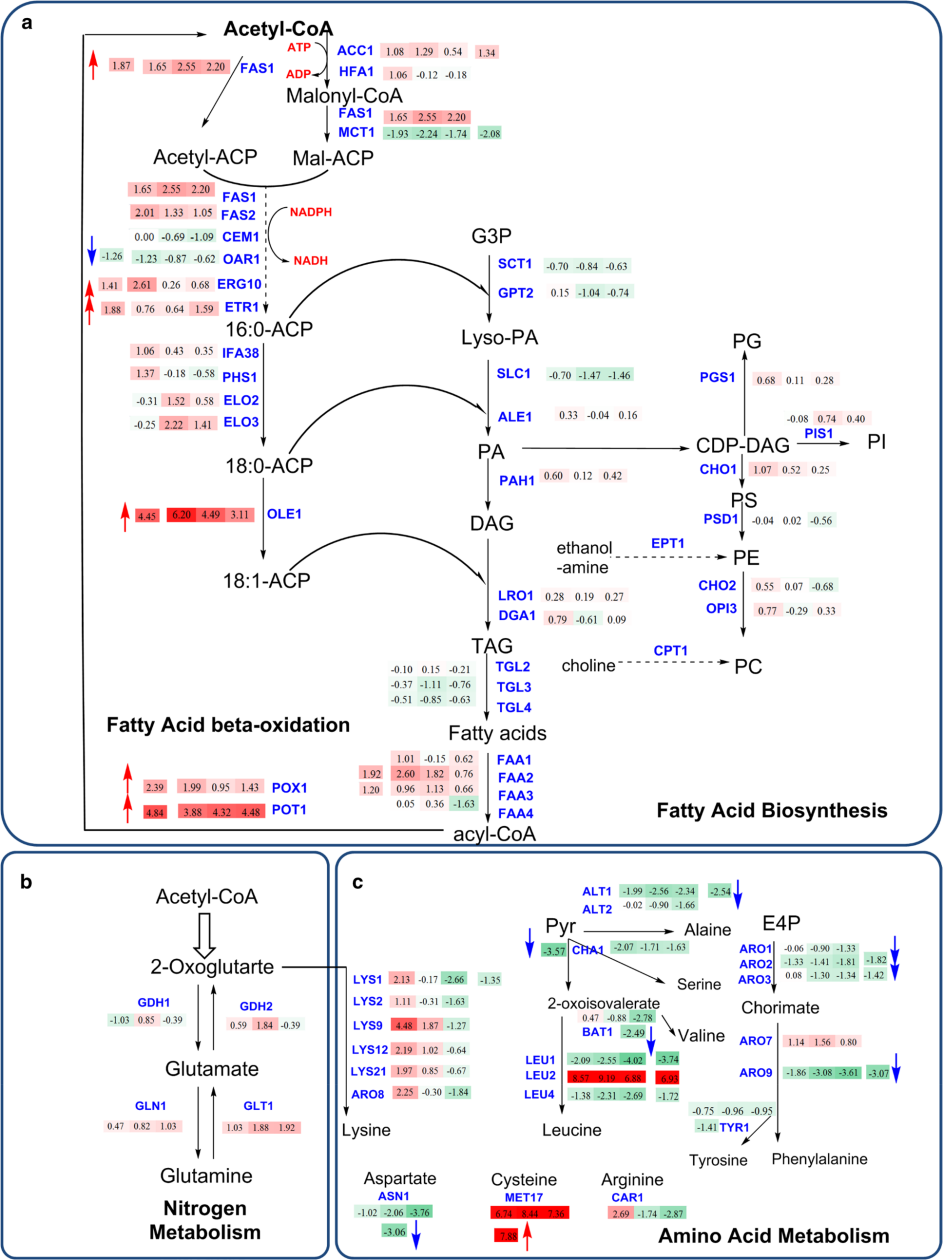
Transcript levels of genes in the metabolic pathways of fatty acid biosynthesis (**a**), nitrogen metabolism (**b**), and amino acid metabolism (**c**) in D5A compared to BY4741. Up-regulated or down-regulated levels are indicated by log_2_-based values in shaded rectangles with red and blue arrows, respectively. Time series changes (T1, T2, and T3) of D5A compared to BY4741 during N deprivation are indicated by shaded rectangle boxes with the log_2_-transformed values from left to right. Shades of green indicate the respective degree of which the gene is down-regulated and shades of red indicate the respective degree of which the gene is up-regulated. All transcriptional differences are shown as the mean of duplicate log_2_-based values

Similar to the transcriptional profiles in D5A during N deprivation, the key genes coding for fatty acid biosynthesis enzymes also showed increased transcription levels in D5A versus BY4741 (Fig. 4), especially *OLE1* which was up-regulated 21.9-fold (Table 3). The increased lipid content in D5A was also correlated closely with increased transcript levels of genes coding for fatty acid biosynthesis enzymes (e.g., FAS1, ERG10, ETR1, and OLE1) by increasing carbon precursors to form more fatty acids.

### TAG and phospholipid turnover in D5A contribute to lipid accumulation

In contrast to transcript levels for fatty acid biosynthesis enzymes, most genes involved in TAG assembly showed down-regulation or no significant difference in D5A during N deprivation or when compared to BY4741 (Figs. 2, 4). TAG and phospholipids were synthesized from phosphatidic acid (PA), which is derived from glycerol-3-phosphate by glycerol-3-phosphate acyl-transferase (SCT1, GPT2) and lyso-phosphatidic acyl-transferase (SLC1 and ALE1), respectively. Subsequently, PA can be dephosphorylated by phosphatidate phosphatase (PAH1) to produce diacylglycerol (DAG) [50]. DAG is then acylated by acyl-CoA-dependent diacylglycerol acyl-transferase (DGA1) or phospholipid-dependent diacylglycerol acyl-transferases (LRO1), respectively. Unexpectedly, all these genes showed down-regulation or no significant difference in D5A during N deprivation or when compared to BY4741 (Figs. 2, 4). Interestingly, the transcript levels of GPT2, SLC1, ALE1, and PAH1 genes were down-regulated more than twofold in different time points during N deprivation in D5A (Fig. 2).

Although most of the aforementioned genes were down-regulated, the lipid content in D5A was enhanced under N deprivation. Such results with increased lipid content and decreased transcript level of TAG biosynthesis genes in D5A seem contradictory. Results from engineering *Nannochloropsis gaditana* strains provided some insights into the mechanisms underlying regulation of lipid accumulation in *N. gaditana* [51]. Similar to our results, they indicated that enzymes for TAG synthesis are present in sufficient quantities before N depletion allowing continued lipid accumulation without significant differences in transcript levels in FAS- and TAG-assembly genes [51].

Another possibility is that TAG oxidation was reduced in N deprivation, resulting in an overall increase in TAG accumulation, even though de novo biosynthesis did not increase significantly. Transcript levels for gene-encoding enzymes needed for the degradation of TAG and fatty acids, including triacylglycerol lipase (TGL), fatty acid-CoA synthase (FAA), and acyl-CoA oxidase (POX1, POT1), were decreased dramatically in **T3** phase in D5A during nitrogen deprivation (Fig. 2). Furthermore, the transcript level of POX1 and FAA1 decreased about 2.7- and 2.6-fold, respectively, for all time points during nitrogen deprivation in D5A (Table 3). Therefore, large amounts of lipids can be retained in the cells rather than degraded through the *β*-oxidation pathway.

In addition, phospholipids constitute the cell membrane, which is essential for cellular integrity [18]. Thus, accumulated lipids act not only as an energy and carbon sink, but also contribute to the increase of phospholipid monolayers for membrane stabilization [52]. In the pathway towards phospholipids, PA is first activated to CDP-diacylglycerol (CDP-DAG) and thereafter incorporated into the different types of phospholipids [22]. Our results indicated that transcript levels for phosphatidylinositol synthase (PIS1), which generates phosphatidylinositol, a precursor for membrane phospholipids, were increased by time point **T3** (Table 3). In contrast, CDP-diacylglycerol-serine *O*-phosphatidyl transferase (CHO1), phosphatidylserine decarboxylase (PSD1), and ethylene-fatty-acyl-phospholipid synthase (OPI3)-encoding enzymes for the biosynthesis of phosphatidylserine, phosphatidylethanolamine, and phosphatidylcholine were down-regulated in different time points during nitrogen deprivation in D5A (Fig. 2). These results suggest that phospholipids may undergo turnover and acyl-chain remodeling for repartitioning to storage lipid biosynthesis [50].

### FA, TAG, and phospholipid turnover differ between D5A and BY4741

Similar to D5A during N deprivation, the transcript levels of genes coding for TAG assembly were also decreased or had no significant difference in transcription levels in D5A compared to BY4741 (Fig. 4). Interestingly, a dramatic increase in transcript levels of FAA, POX1, and POT1 was found in D5A compared to BY4741 during N deprivation (Fig. 4). It seems more likely that the overall flux toward FA biosynthesis in D5A is higher than that for fatty acid utilization via *β*-oxidation compared with BY4741. Such inconsistency of high levels of gene expression between lipid accumulation and *β*-oxidation is also found in the oleaginous yeast, *Y. lipolytica* [42]. There was no significant difference in transcript levels in phospholipid biosynthesis genes between D5A and BY4741 (Fig. 4), indicating that phospholipid conversion may not be the reason for higher lipid content in different strain backgrounds.

These results thus suggested that different mechanisms may exist for lipid accumulation in different strain backgrounds. The increased lipid content in D5A during N deprivation could be ascribed to the increase of fatty acid biosynthesis, reduced TAG degradation, as well as phospholipid turnover for TAG accumulation.

### Alternative nitrogen metabolism increases lipid accumulation in D5A

Glutamine synthetase and glutamate synthase routes are usually used for nitrogen assimilation in bacteria, photosynthetic algae, and higher plants [34, 45]. Nitrogen assimilation occurs via two routes: (1) glutamate dehydrogenase (GDH1), which is NADP^+^-dependent and utilizes NADP^+^ to convert *α*-ketoglutarate and NH_3_ to glutamate; or (2) glutamine synthase (GLN1), which produces glutamine from glutamate and NH_3_ with energy from ATP [53]. Nitrogen from the catabolism or redistribution from proteins and non-essential amino acids during nitrogen stress is stored as essential amino acids and intermediate molecules via metabolic pathways such as the glutamate–glutamine pathway [45], which may play a role in lipid metabolism in oleaginous microorganisms [9].

Genes involved in nitrogen and amino acid metabolism were up-regulated in D5A under N deprivation (Fig. 2). In nitrogen metabolism, N assimilation occurs via the glutamine synthetase and glutamate synthase pathways using GDH1, GLN1, NADH-dependent glutamate synthase (GLT1), and NAD^+^-linked glutamate dehydrogenase (GDH2) [54]. GDH1 catalyzes glutamate formation from *α*-ketoglutarate, whereas GLN1 is usually up-regulated for nitrogen assimilation during N starvation as reported previously in other organisms [34, 55]. GDH2 drives the production of *α*-ketoglutarate from glutamate, and GLT1 catalyzes the formation of glutamate from glutamine [34, 53]. The transcript level of these four nitrogen metabolism genes progressively increased at three time points with significant changes in D5A (except for GDH2 in **T3**) (Fig. 2), which are closely coupled to a “metabolic hub” centered around glutamate. When N is assimilated into glutamate and glutamine, amino-transferases redistribute it to other molecules, including amino acids. Once N is insufficient, cellular protein is usually degraded and N can be recycled to amino acids to mitigate stress [45]. Transcript levels of *GDH1*, *GLN1*, *GLT1*, and *GDH2* were found to be up-regulated by 2.7-, 3.6-, 3.0-, and 1.5-fold, respectively (Table 3). N assimilation plays an important role in lipid metabolism, and the increase of these amino acids, like glutamate and glutamine, makes an important contribution to lipid biosynthesis.

For amino acid metabolism, the transcript levels of most genes were dramatically up-regulated in D5A during N deprivation at **T1** and **T2**, but decreased at **T3** without significant difference compared to the N-repleted condition (Fig. 2). Under environmental stress, de novo protein synthesis is generally inhibited, and the protein turnover rates are increased. Nitrogen was possibly recycled from protein degradation to amino acids to mitigate stress when confronting N depletion [45]. Thus, transcript levels of amino acid metabolism would have peaked in **T1** or **T2**. Decreased levels in **T3** may help redirect carbon flux from these amino acids to lipids by providing carbon skeletons like acetyl-CoA for fatty acid elongation. For example, the catabolism of alanine and lysine can provide the carbon skeletons directed to fatty acid elongation [56]. Gene-encoding *β*-isopropylmalate dehydrogenase (LEU2) and branched-chain amino acid (BCAA) aminotransferase (BAT1) were greatly up-regulated in **T2** and **T3** (Fig. 2). These two genes also increased about 5.4- and 9.8-fold in D5A under N deprivation (Table 3). They were closely related to the metabolism of leucine, which has been suggested to be involved in the regulation of lipid metabolism in the oleaginous yeast, *Y. lipolytica* [36]. Similarly, increased leucine levels may also be sensed in D5A and as a transcriptional response further down-regulates amino acid biosynthesis at **T3**, thus providing stronger metabolic flux towards lipid metabolism. In addition, genes responsive for allantoin utilization (*DUR12* and *DAL1*) were found to be up-regulated, which represents the cellular redirection of nitrogen under N deprivation. Overall, the metabolic network is adapted to divert the carbon flux from pathways requiring nitrogen, such as amino acid metabolism, to lipid biosynthetic pathways.

When comparing D5A with BY4741, N assimilation genes of *GLN1* in **T3**, *GDH2* in **T2**, and *GLT1* from **T1** to **T3** were up-regulated significantly during N deprivation (Fig. 4), whereas no significant difference was found between the two strains without time consideration. It seems likely that nitrogen is preferred to be assimilated into glutamate, which is the central hub in carbon and nitrogen metabolism. The increase of glutamate enhances the activity of the TCA cycle enzymes to supply biosynthetic building blocks [47]. Interestingly, the majority of genes involved in amino acid metabolism were greatly decreased in three time points or only up-regulated in the initial phase, except for *LEU2* and *MET17* (required for methionine and cysteine biosynthesis) which increased dramatically (Fig. 4). Down-regulation of multiple amino acid metabolism genes appears to restrict metabolic flux to amino acids and protein synthesis, thus resulting in the allocation of carbon into storage lipids in D5A. Significant up-regulation of *LEU2* (121.9-fold) in comparison of strains (Table 3) can explain the lipid accumulation in D5A, but not in BY4741, due to the role of leucine metabolism in lipid biosynthesis. Amino acids are important building blocks of proteins and serve as the precursors of N-containing molecules, such as nucleic acids, polyamines, quaternary ammonium compounds, and some hormones [45]. The previous studies found that the addition of leucine and methionine to a prototrophic Δ*SNF1* strain increased its growth rate, whereas having no effect on the isogenic wild-type strain [47], suggesting a more complex role for SNF1, which includes regulating amino acid metabolism.

### Regulation of central carbon metabolism increases lipid biosynthesis

#### Carbon precursors

The Embden–Meyerhof–Parnas (EMP) pathway provides an additional conversion of carbon to lipids in D5A during N deprivation. Glucose was utilized to generate pyruvate in the cytoplasm through the EMP pathway. Most genes in the EMP pathway were affected immediately in **T1** (Fig. 3), corresponding to the slower glucose consumption rate during N stress (Additional file 1: Figure S1). After a period of adaption to N deprivation in the initial stage of **T1**, the transcriptional levels of these genes were gradually increased in **T2** and **T3**, especially genes coding for fructose 1,6-bisphosphatase aldolase 1 (FBA1), hexokinase 2 (HXK2), and enolase II [a phosphopyruvate hydratase (ENO2)]. Nonetheless, from an overall perspective without the consideration of each time point, the expression of EMP genes was still decreased significantly, except for HXK2, which increased for 7.3-fold (Table 3). The EMP pathway is the major contributor for providing pyruvate and acetyl-CoA flux for fatty acid biosynthesis. Thus, long-term repression of EMP will limit lipid accumulation [33]. Therefore, the EMP pathway genes gradually increased in **T2** and **T3** may help to convert more carbon into lipid through acetyl-CoA in D5A. Moreover, fatty acid biosynthesis is an energy-intensive process. ATP is the main energy source and the ATP/AMP ratio has been found to be increased in yeast cell during lipid biosynthesis, which can also be generated from EMP [14].

Decreased production of ethanol was found in D5A during N deprivation. Gene-encoding alcohol dehydrogenases (ADHs) play an important role in the ethanol fermentation. In *S. cerevisiae*, genes of classical ADHs include: *ADH1*, *ADH2*, *ADH3*, *ADH4*, and *ADH5* [57]. Among them, *ADH1* is mainly responsible for the production of ethanol from acetaldehyde in cells grown anaerobically or in the presence of a glucose excess, whereas *ADH2* is repressed under these conditions and primarily functions to convert ethanol accumulated when cells are grown on respiratory carbon sources [57, 58]. The *ADH3*, *ADH4,* and *ADH5* genes play roles in producing ethanol from acetaldehyde [57]. Most transcript levels of these ADHs were down-regulated by the lipid accumulation phase in D5A during N deprivation (Fig. 3), and decreased ethanol production from **T2** was also observed (Additional file 1: Figure S1). The previous studies indicated that the inactivation of *ADH1* helps to redirect the metabolic flux from ethanol production to fatty acids synthesis [46]. In addition, the over-expression of *ADH2* and acetaldehyde dehydrogenase (*ALD6*) which converts ethanol to acetate, together with the heterologous gene-encoding acetyl-CoA synthetase, resulted in a threefold improvement of fatty acid ethyl ester content [59]. Contrary to our expectation, no significant changes and even decreased transcript levels (2.6-fold decrease at **T1** in *ALD6*) were found for these genes. However, the transcript level of *ADH4* decreased significantly from **T1** to **T3** (Fig. 3). Overall, the reduced ethanol production observed in D5A suggests the redirection of metabolic flux from ethanol to fatty acid biosynthesis.

Acetate from ethanol oxidation could be partitioned into acetyl-CoA catalyzed by acetyl-CoA synthetase (ACS1 and ACS2) [60]. The transcript level of ACS1 decreased greatly from **T2** to **T3**, whereas ACS2 was dramatically up-regulated in **T3** (Fig. 3). ACS2 is believed to be mainly cytosolic and ACS1 is believed to be mainly peroxisomal [12]. Thus, ACS2 could likely contribute to the rapid conversion of ethanol to the native acetyl-CoA supply in the cytosol. In addition, glycerol, which is often involved in cellular homeostasis responding to osmolarity and acts as a redox valve to dispose of excess cytosolic NADH during exponential growth [47], gradually increased in D5A during N deprivation (Additional file 1: Figure S1). More importantly, genes coding for glycerol-3-phosphate phosphatase (GPP1 and GPP2) in glycerol degradation were dramatically up-regulated in **T2** and **T3** (Fig. 3), which can provide glycerol-3-phosphate for fatty acid biosynthesis [34].

The transcript levels of genes in the EMP pathway, as well as genes for ethanol and glycerol production were up-regulated in D5A versus BY4741 (Fig. 6). The physiological results of rapid glucose consumption under both nitrogen concentrations in D5A (Additional file 1: Figure S1) corresponded to the increase of EMP pathway genes, especially *HXK1* and *ENO1*. This finding suggests that D5A possess excellent carbon fixation and utilization potential compared to BY4741. More remarkable, ethanol production genes were initially up-regulated in **T1**, but deceased in the following time points, whereas the ethanol degradation genes of *ADH2* and *ALD6*, as well as the ACS genes, were greatly up-regulated in D5A (Fig. 6). These changes at the transcriptional level corresponding to decreased ethanol production in D5A (Additional file 1: Figure S1) support the hypothesis that D5A can divert acetyl-CoA destined for ethanol production to fatty acid biosynthesis. For glycerol metabolism, even though the transcriptional level of most genes was up-regulated, the glycerol content in D5A did not exceed the glycerol content in BY4741 (Additional file 1: Figure S1). It is possible that post-transcriptional modification may be involved in glycerol metabolism regulation. For example, in nutrient-limited conditions, the major glycerol-3-phosphate dehydrogenase activity was phosphorylated and inactivated by SNF1 [47].

#### Reducing equivalents

The TCA cycle plays a pivotal role in utilizing non-fermentable carbon sources via oxidative generation of reducing equivalents (NADH) driving aerobic respiration to yield ATP [61]. In D5A, during N deprivation, the transcript levels of almost all TCA cycle genes were down-regulated (Fig. 3). In general, fatty acid biosynthesis can be promoted by the enhanced activity of the TCA cycle under N starvation in oleaginous yeasts [62], microalgae, and plants [63], due to its function as an essential component providing acetyl-CoA and NADH in cells [56, 62, 64]. However, *S. cerevisiae* could not use the acetyl-CoA from the TCA cycle due to the lack of ATP-citrate lyase (ACL) [14, 65]. Interestingly, three genes of carnitine acetyltransferases (CAT2, YAT1, and YAT2) mediating the translocation of cytosolic acetyl-CoA into mitochondria from the TCA cycle [61] were dramatically down-regulated, especially YAT1 (> 16.8-fold) (Table 3). Thus, migration of acetyl-CoA toward the TCA cycle was blocked and remained in the cytosol to be directly channeled into lipid biosynthesis.

The reducing equivalents supply that should be provided from the TCA cycle may be replaced by the alternative glyoxylate cycle, which converts acetyl-CoA to four-carbon dicarboxylic acids bypassing oxidative decarboxylation accompanied with the generation of NADH [61]. The up-regulation of malate synthase enzyme (MLS1), cytoplasmic malate dehydrogenase (MDH2), and isocitrate lyase (ICL1) in **T1** suggests a compensatory mechanism involving NADH biosynthesis (Fig. 3). It is well known that fatty acid elongation requires NADPH, which can be produced via pentose phosphate pathway (PPP) in the cytosol of *S. cerevisiae* by glucose-6-phosphate dehydrogenase (ZWF1) and phosphogluconate dehydrogenase (GND) [14, 59]. Although *ZWF1* did not increase at any time points (Fig. 3), *GND1* was up-regulated in D5A in **T2** and **T3**. Notably, 6-phosphogluconolactonase (SOL3) and transketolase (TKL1) were also greatly up-regulated in D5A under N deprivation (Table 3). Oxidative PPP is important for supplying NADPH to the cytoplasm for fatty acid biosynthesis, which has been reported in *R. toruloides*, *L. starkeyi,* and *Y. lipolytica* [52, 62, 66, 67]. In addition, the increased lipid content may feedback to the glucose-6-phosphate (G6P) node by accelerating NADPH oxidation, resulting in G6P conversion through the oxidative PPP to meet the NADPH demand [62].

Similar to the pathways providing carbon precursors, pathways providing reducing equivalents were also up-regulated in D5A versus BY4741. It is reasonable that the up-regulation of TCA cycle genes is closely related to elevated respiration in anaerobic *S. cerevisiae* cultures, which resulted in rapid growth and promoted fatty acids biosynthesis in D5A. Although the building blocks of acetyl-CoA cannot be provided, the supply of reducing equivalent is equally important for lipid biosynthesis in *S. cerevisiae*. PPP and glyoxylate cycle genes were up-regulated to provide sufficient reducing power for rapid growth and lipid accumulation in D5A versus BY4741.

Taken together, D5A exhibited enhanced central carbon metabolism over BY4741, thus, providing large amounts of carbon and energy flux toward lipid accumulation.

### Potential regulators involved in lipid biosynthesis

**Fig. 5:**
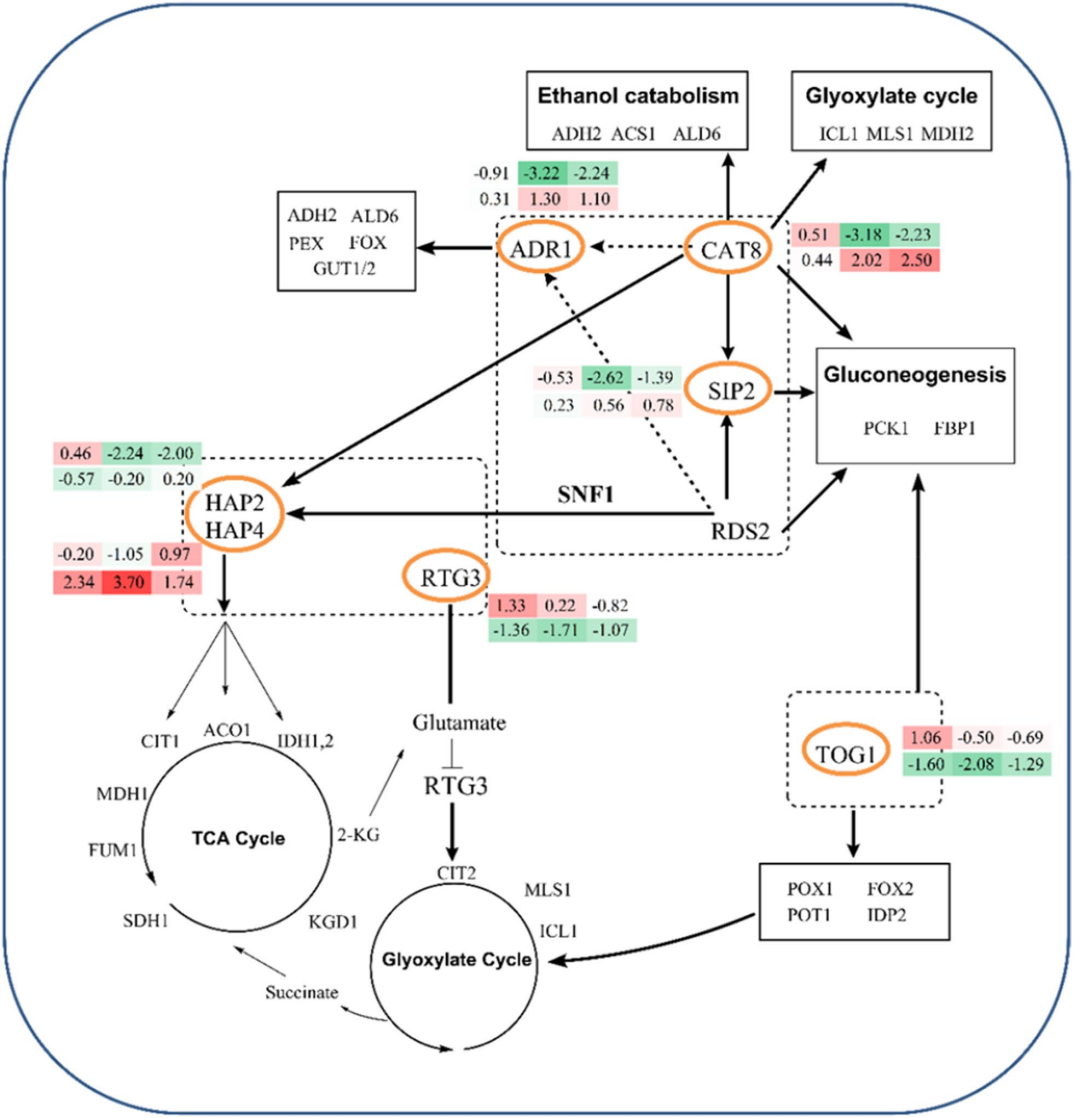
Transcript levels for transcriptional regulators related to carbon and nitrogen metabolism. The numbers in the two rows of shaded rectangles next to orange circled gene names indicate the log_2_-transformed fold changes in time points T1, T2, and T3, from left to right, respectively. The top row represents transcriptional differences in D5A during N deprivation [5 mM (NH_4_)_2_SO_4_, − N] compared to N replete [35 mM (NH_4_)_2_SO_4_, + N]. The bottom row represents transcriptional differences between the D5A and BY4741 strains. All transcriptional differences are shown as the mean of duplicate log_2_-based values. The gene names in the boxes show the pathway genes affected by the differentially expressed regulators

The expression profiles of a few regulatory genes closely related to lipid biosynthesis metabolic networks at different oleaginous phases and conditions were also investigated (Fig. 5). As previously mentioned, *SNF1* is an important regulator controlling the induction of genes involved in gluconeogenesis, glyoxylate cycle, amino acid utilization, as well as general stress responses [16, 32, 47]. However, there are no significant differences at the transcriptional level in this study, and the role of *SNF1* may be at the post-transcriptional level as discussed above.

For carbon metabolism, several lipid biosynthesis-related regulators, including ADR1, CAT8, SIP2, and HAP2, were significantly down-regulated, but TOG1 was up-regulated in D5A during N deprivation. First, the biochemical functions of proteins regulated by CAT8 were involved in the first step of ethanol utilization, glyoxylate cycle, and gluconeogenesis [68, 69]. The transcript level of CAT8 was significantly decreased (especially in **T2** and **T3** time points), which corresponds to the down-regulation of gluconeogenic and glyoxylate cycles in D5A under N stress (Fig. 5). Second, SIP2 and ADR1, which are involved in the control of gluconeogenesis; as well as ethanol, glycerol, and fatty acid utilization during glucose exhaustion [69], were down-regulated in D5A under N deprivation mainly in **T2** and **T3**. Third, HAP genes (HAP2/3/4/5) are known to be involved in respiratory gene expression. HAP2 significantly decreased in D5A under N stress, which corresponded to decreased transcript level of the TCA cycle genes. In comparison, HAP4 was greatly increased for 6.0-fold in strain comparisons (Fig. 5). Undoubtedly, respiratory genes in the TCA cycle were also up-regulated in D5A versus BY4741. Fourth, TOG1 is an important activator for oleate utilization. Its transcript level increased in **T1**, but decreased in **T2** and **T3** in D5A during N deprivation (Fig. 5). Down-regulation of TOG1 could reduce transcript levels of genes involved in fatty acid *β*-oxidation (*POX1*, *FOX2*, and *POT1*), NADPH regeneration (*IDP2*), the glyoxylate shunt (*MLS1* and *ICL1*), and gluconeogenesis (*PCK1* and *FBP1*) [69]. Thus, fatty acid production can be retained and more carbon can be provided for its biosynthesis in D5A under N deprivation.

Carbon and nitrogen metabolism are closely related. For nitrogen metabolism, genes involved in converting oxaloacetate to *α*-ketoglutarate in TCA cycle are controlled by the retrograde (RTG) pathway [54]. Intracellular glutamate and its precursor *α*-ketoglutarate are important for the RTG activators. The transcript level of RTG3 showed up-regulation in **T1** and **T2**, but decreased in **T3** in D5A during N deprivation (Fig. 5).

**Fig. 6:**
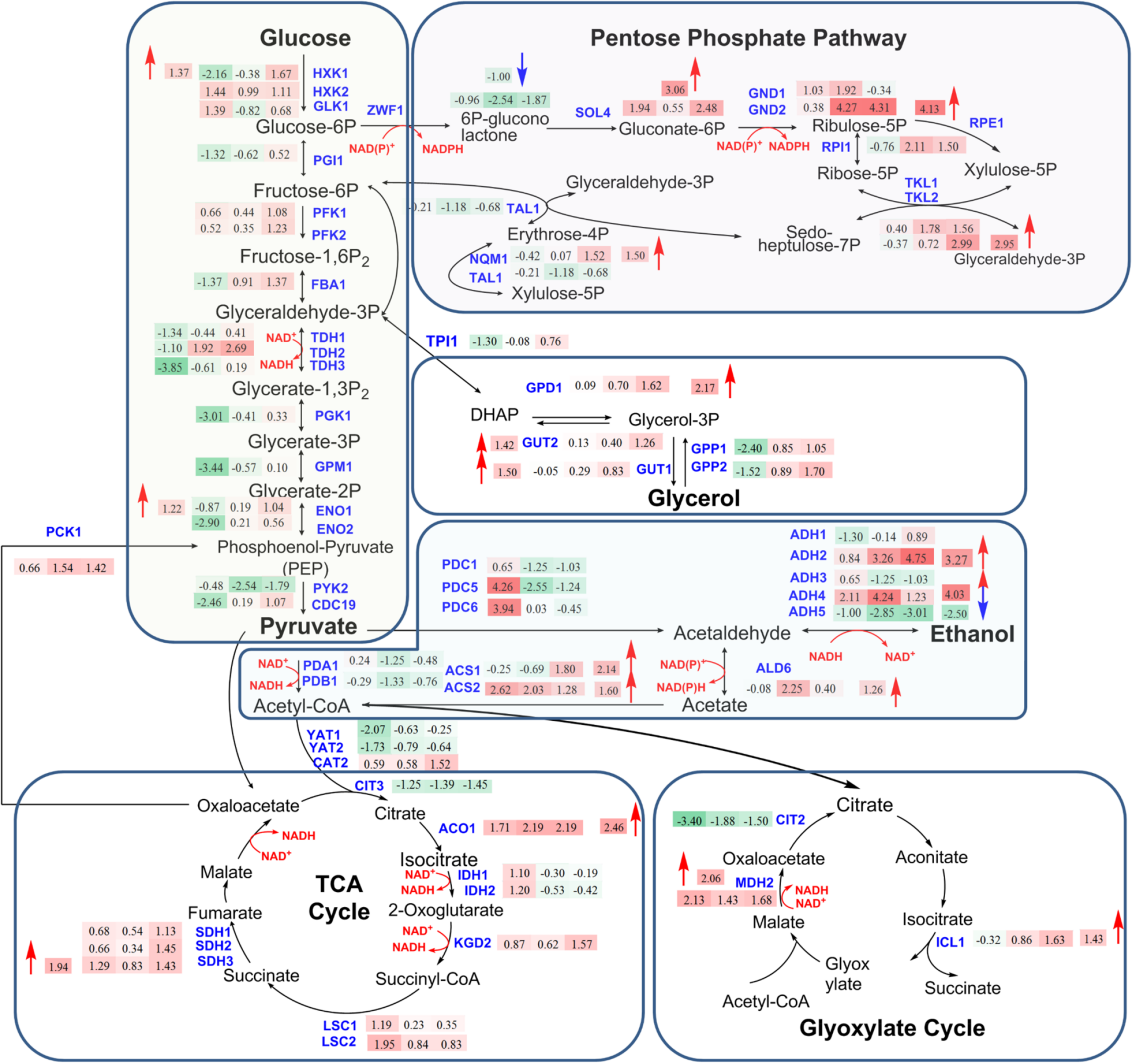
Transcript levels of genes in central carbon metabolism in D5A compared to BY4741. Up-regulated or down-regulated levels are indicated by log_2_-based values in shaded rectangles with red and blue arrows, respectively. Time series changes (T1, T2, and T3) of D5A compared to BY4741 during N deprivation are indicated by shaded rectangle boxes with the log_2_-transformed values from left to right. Shades of green indicate the respective degree of which the gene is down-regulated and shades of red indicate the respective degree of which the gene is up-regulated. All transcriptional differences are shown as the mean of duplicate log_2_-based values

In comparing the two strains, CAT8 was up-regulated 3.2-fold in D5A compared to BY4741 and could contribute to the oleaginicity of D5A (Fig. 5), which was supported by the increased transcript levels of *PCK1*, *MDH2*, and *ICL1*; as well as genes for ethanol catabolism (*ADH2*, *ALD6*, and *ACS1*) (Fig. 6). No significant difference was found for the regulators of SIP2 and ADR1. TOG1 was greatly down-regulated in D5A versus BY4741 (3.14-fold). The regulated genes involved in fatty acid *β*-oxidation, NADPH regeneration, glyoxylate shunt, and gluconeogenesis were also decreased (Table 3). The transcript level of RTG3 significantly decreased in strain comparisons (Fig. 5). It repressed the transcription of RTG-regulated genes in D5A under N limitation, including genes such as *CIT1/2*, *ACO1*, and *IDH1*,*2* (Table 3), which are involved in converting oxaloacetate to *α*-ketoglutarate in the TCA cycle [54]. This repression decreased the concentration of the intracellular *α*-ketoglutarate, the precursor of glutamate and glutamine, and, finally, reduced the concentrations of intracellular amino acids. Therefore, the deceased amino acids, as well as N metabolism had greater effect on D5A than on BY4741.

Regulators discussed above are closely related to carbon and nitrogen metabolic pathway genes which are important for lipid utilization and accumulation. More work is still needed though to completely unravel the regulatory mechanisms of lipid accumulation in D5A.

### D5A and other typically oleaginous organisms use different lipid accumulation pathways

Yeast species such as *R. glutinis*, *L. starkeyi,* and *Y. lipolytica*, and microalgae accumulate large amounts of lipid when these oleaginous organisms are subject to nutrient shortages [9, 15, 33, 36, 70, 71]. Thus, we further explore the differences between D5A and these oleaginous organisms from the following three aspects: (1) de novo lipid biosynthesis, (2) carbon precursors, and (3) reductant equivalents.

First, although *DGA1* and *LRO1* were up-regulated in other oleaginous microorganisms or plants [48, 64], there were no significant differences for essential TAG biosynthesis genes such as *DGA1, SLC1,* and *LRO1* in D5A. Therefore, the accumulation of TAG in D5A seems to be a consequence of the reallocation of carbon and reductant equivalents by a “push” strategy to increase the concentrations of upstream fatty acids through the up-regulation of lipid biosynthesis genes such as *ELO* and *OLE1*, rather than a “pull” strategy through the up-regulation of TAG biosynthesis genes.

Second, ACL, an enzyme unique to oleaginous yeasts for lipogenesis, is absent in *S. cerevisiae*. In oleaginous yeasts, nitrogen exhaustion decreases the concentration of intracellular adenosine monophosphate (AMP), resulting in the inhibition of isocitrate dehydrogenase, which leads to the accumulation of citrate in the mitochondrion [13]. ACL actively participates in cleaving the citrate formed in the mitochondria via the TCA cycle with acetyl-CoA transported into the cytosol for lipid accumulation [14, 65]. Acetyl-CoA is then directed to the de novo FA biosynthesis pathway. However, in D5A, acetyl-CoA generation cannot be provided by citrate from the TCA cycle. The decreased transcript level of carnitine acetyltransferases discussed above is a way to retain acetyl-CoA in the cytosol, and the diversion of acetyl-CoA away from ethanol production could be another major precursor source for FA biosynthesis and lipid accumulation in *S. cerevisiae* D5A.

Third, the supply of reducing equivalents including NADPH or NADH for lipid biosynthesis can also be provided from different sources, including the malic enzyme (ME), PPP, or the NADP^+^-dependent isocitrate dehydrogenase [15]. Although the malic enzyme is considered the major NADPH source in many oleaginous fungi for lipid biosynthesis [14, 72], it does not play an active role (no significant difference was found) in D5A. Similar results were also reported in *Y. lipolytica* [62]. Alternatively, oxidative PPP is the primary source of NADPH required for lipid biosynthesis in D5A, which is consistent with *Y. lipolytica* [62] and presumably in *R. toruloides* [73].

Similar changes at the transcriptional level were also found in D5A and different oleaginous microorganisms for genes in other metabolic pathways. For example, the up-regulation of N assimilation genes, such as *GLN1* and *GLN2,* and the role of leucine metabolism during N deprivation in D5A are consistent with that in *Y. lipolytica* [34, 36].

The enhanced expression of enzymes involved in carbon utilization, ethanol conversion, and TCA cycle in D5A contributes greatly to its lipid accumulation versus BY4741. The genetic roles of the native characteristics of D5A are important, such as rapid glucose consumption and high ethanol production in **T1** time point. Accordingly, lipid accumulation in *S. cerevisiae* D5A can be increased by enhancing or restricting the N assimilation pathway or C metabolism via genetic manipulation for the efficient production of cytosolic acetyl-CoA and NADPH. In addition, the over-expression of key genes in the TAG biosynthesis pathway and the alternative route to divert carbon flux from ethanol towards acetyl-CoA synthesis could further promote lipid accumulation. Many intracellular processes are involved in sophisticated regulatory mechanisms for lipid accumulation, not only at the transcriptional level, but also at the levels of post-transcriptional modification and metabolic flux regulation. All these processes may be integrated to regulate the lipid accumulation potential of D5A. Although the over-expression of genes in fatty acid biosynthesis have been carried out before [59], the complicated interplay among genes still needs to be explored further, and new genome editing tools for strain improvement to improve lipid accumulation are needed, which have been successfully applied in the oleaginous microalgae *N. gaditana* [51].

## Conclusion

In this study, the transcriptional profiles of lipid accumulation in an oleaginous yeast *S. cerevisiae* D5A under different nitrogen conditions were investigated and the underlying mechanism of the oleaginicity of D5A was proposed. Unlike typical *S. cerevisiae*, the D5A strain accumulates high levels of lipids during N deprivation and the combined coordinated responses of fatty acid precursor biosynthesis, nitrogen metabolism, PPP, ethanol degradation, and leucine metabolism led to this lipid accumulation. During N deprivation, decreased fatty acid *β*-oxidation, phospholipid remodeling, and reducing equivalent compensation from glyoxylate cycle and carbon–nitrogen disequilibrium further relocated acetyl-CoA and NADPH into the cytosol for lipid biosynthesis. In addition, the EMP and TCA cycle were enhanced in D5A, which facilitated carbon diversion to lipid accumulation in aerobic conditions. Transcriptional regulators within the cellular regulatory network governing carbon, nitrogen, and lipid metabolism are involved in lipids biosynthesis regulation in D5A. Our result demonstrated that *S. cerevisiae* D5A is a potential candidate strain for industrial lipid production due to its rapid growth, high lipid content, and ease of genetic engineering.

## Methods

### Strains and growth condition

The *S. cerevisiae* D5A (ATCC 200062) and BY4741 (ATCC 201388) strains were grown in YPD medium containing 10 g/L yeast extract (Merck Millipore, MA,), 20 g/L peptone (Difco, VWR, Stockholm, Sweden), and 50 g/L glucose at 28 °C, 200 rpm. Two concentrations of (NH_4_)_2_SO_4_, 5 mM (nitrogen deprivation, − N) and 35 mM (NH_4_)_2_SO_4_ (nitrogen repletion, + N), were provided in triplicate for fermentation.

The growth of *S. cerevisiae* cultures was followed by measuring the optical density at 600 nm in a model 8453 spectrophotometer (Hewlett-Packard, CA). Samples were harvested at 10 h, 25 h, and 70 h post-inoculation in strain D5A and BY4741. These three sampling time points for each strain can also be described as initial growth phase (**T1**), lipid accumulation phase (**T2**), and late oleaginous phase (**T3**), respectively. The cells were harvested by centrifugation at 1000×*g* for 5 min. The cells were washed once with 5 mL phosphate buffer (10 mM KH_2_PO_4_, pH 7.5). The supernatant was removed; the pellet was frozen in liquid nitrogen and freeze dried (Christ Alpha 2-4 LSC, Martin Christ Gefriertrocknungsanlagen GmbH, Osterode am Harz, Germany) for 48 h.

### FAME content analysis

The cell biomass was harvested by centrifugation and freeze dried prior to FAME determination. In brief, 0.2 mL chloroform–methanol (2:1 v/v) was added to 7 mg to 11 mg of lyophilized biomass to solubilize the lipids and simultaneously transesterify the lipids in situ with 0.3 mL HCl–methanol (95% v/v) for 1 h at 85 °C in the presence of tridecanoic acid (C13) methyl ester as an internal standard. The resulting FAMEs were extracted with 1 mL hexane at room temperature for 1 h and analyzed by gas chromatography GC-FID (Agilent 6890 N) using HP5-MS column (Agilent, USA), 30 m × 0.25 mm internal diameter × 0.25 μm (FT). Hexane extracts were injected at 1 μL, 10:1 split ratio, and an inlet temperature of 250 °C. A constant flow rate of 1 mL/min helium was maintained with an oven ramping program starting at 70 °C, 20 °C/min to 230 °C, hold 1 min; 20 °C/min to 325 °C, hold for 5 min. Detector temperature was set at 325 °C, 40 mL/min hydrogen, 400 mL/min zero air, and 20 mL/min helium makeup gas. Quantification of the FAMEs was based on the integration of individual fatty acid methyl ester peaks in the chromatograms and quantified using a five-point calibration curve of a mixed, even carbon-chain FAME standard of 14 individual fatty acids (C8–C24, SIGMA cat# 18918), and normalized for the C13 methyl ester internal standard recovery. The total FAME content was calculated as the sum of the even numbered FAMEs’ contributions. All samples were analyzed in triplicates, and the standard deviations were < 0.35%.

### High-performance liquid chromatography (HPLC)

HPLC analysis was used for the measurement of the concentrations of glucose, xylitol, acetate, and ethanol within 0.2 μm-filtered samples taken at different time points during fermentation (Additional file 1: Figure S1) and analyzed as described previously [74]. The diluted fermentation samples (1:1 with 8.98 mM sulfuric acid) were separated and quantified by HPLC using a LaChrom Elite System (Hitachi High Technologies America, Inc., San Jose, CA). Analysis was performed with an oven (Model L-2350) set at 60 °C, and a pump (Model L-2130) set with a flow rate of 0.5 mL/min in 5 mM H_2_SO_4_. The run time for each sample was set for 35 min (Injector Model L-2200). Eluted compounds were registered and quantified by a refractive index detector (Model L-2490) equipped with a computer-powered integrator. Soluble fermentation products were identified by comparison with retention times and peak areas of corresponding standards. Metabolites were separated on an Aminex HPX-87H, 300 mm × 7.8 mm column (Bio-Rad, Hercules, CA).

### Total RNA extraction, RNA-Seq, and data statistical analysis

Samples from the fermentation with two nitrogen concentrations [5 mM and 35 mM (NH_4_)_2_SO_4_] and three time points (**T1**, **T2,** and **T3**) for strains D5A and BY4741 with biological replicate data were used for statistical analysis to understand the transcriptomic profiling. Samples were immediately centrifuged for 2 min at 25,000×*g*, flash-frozen in liquid nitrogen, and stored at − 80 °C. Total RNA was extracted with Plant RNA Reagent, following the manufacturer’s instructions (Life Technologies, NY). RNA was further purified using a Turbo DNA-free kit (Life Technologies, NY) and a Qiagen RNeasy Plant Mini Kit (Qiagen, CA), followed by checking of the sample quality and quantity with gel electrophoresis and a NanoDrop spectrophotometer (Thermo Scientific, DE). Samples of total RNA were sent to the Genewiz for RNA-Seq using Illumina HiSeq 2000 platform according to the manufacturer’s instructions. Briefly, mRNA was selected using oligo (dT) probes and then fragmented. cDNA was synthesized using random primers, modified, and enriched for attachment to the Illumina flow cell. The fluorescent image process to sequences, base calling, and quality value calculation were performed by the Illumina data processing pipeline (version 1.9).

The gene expression level was indicated by the log_2_-transformed RPKM value. For the purpose of clarity, RPKM mentioned throughout the paper refers to the log_2_-transformed RPKM unless otherwise noted. JMP Genomics was used for statistics analysis as previously described [74]. Briefly, a distribution analysis and data correlation analysis were conducted as a quality control step. The overlaid kernel density estimates derived from the distribution analysis allowed the visualization of sources of variation based on strain and treatment. Data were subsequently normalized, and an analysis of variance (ANOVA) was performed to determine differential expression levels between strains and oleaginous phases using the FDR testing method (*p* < 0.05). The significantly differentially expressed gene lists for different comparisons were generated for further analysis.

## Additional files

**Additional file 1: Figure S1.** Glucose consumption in two strains under different nitrogen concentrations (A) and metabolite production in D5A (B) and in BY4741 (C). Data shown as the mean ± standard deviation of duplicate samples.

**Additional file 2: Table S1.** Next-generation sequence (NGS) results of each sample for *S. cerevisiae* D5A and BY4741.

**Additional file 3: Table S2.** Transcriptomics profiling difference in D5A and BY4741 at different time points and nitrogen concentrations. The ratio is a log_2_-based value. All transcriptional differences are shown as the mean of duplicate log_2_-based values.

**Additional file 4: Table S3.** Differentially expressed genes in the two strains and two nitrogen concentrations at the different time points (T1, T2, and T3). The ratio is a log_2_-based value. All transcriptional differences are shown as the mean of duplicate log_2_-based values.

**Additional file 5: Table S4.** Differentially expressed genes involved in the different pathway including EMP, PPP, FA biosynthesis, TAG biosynthesis, phospholipid biosynthesis, TCA cycle, N and AA metabolism in two strains, and two nitrogen concentrations at different time points (T1, T2, and T3). The ratio is a log_2_-based value. All transcriptional differences are shown as the mean of duplicate log_2_-based values.

## Abbreviations

ACC1: acetyl-CoA carboxylase
ACO1: aconitate hydratase
malonyl-ACP: malonyl-acyl-carrier protein
ACS: acetyl-coA synthetase
ADH: alcohol dehydrogenase
ALD: aldehyde dehydrogenase
ALT: alanine transaminase
ARO8: aromatic aminotransferase I
ASN1: asparagine synthase
BAT1: branched-chain amino acid transaminase
CAR1: arginase
CEM1: mitochondrial beta-keto-acyl synthase
CDC19: pyruvate kinase
CHA1: l-serine/l-threonine ammonia-lyase
CHO1: CDP-diacylglycerol-serine *O*-phosphatidyltransferase
CHO2: phosphatidylethanolamine *N*-methyltransferase
CIT: citrate (Si)-synthase
CPT1: diacylglycerol cholinephosphotransferase
DAK: dihydroxyacetone kinase
DGA1: diacylglycerol acyl-transferase
ENO2: phosphopyruvate hydratase
EOL: elongase
EPT1: sn-1,2-diacylglycerol ethanolamine- and cholinephosphotranferase
ERG10: acetyl-CoA C-acetyltransferase
ETR1: 2-enoyl thioester reductase
FAA: fatty acid-CoA synthase
FAS: fatty acid synthase
FBA1: fructose-bisphosphate aldolase
FBP1: fructose 1,6-bisphosphate 1-phosphatase
FUM: fumarase
GCY1: glycerol 2-dehydrogenase
GDH: glutamate dehydrogenase
GLN1: glutamine synthetase
GLT1: glutamate synthase
GND1, GND2: phosphogluconate dehydrogenase
GPM1: phosphoglycerate mutase
GUT1: glycerol kinase
HFA1: mitochondrial acetyl-CoA carboxylase
HXK: hexokinase
IDH: isocitrate dehydrogenase (NAD)
IDP2: isocitrate dehydrogenase (NADP)
IFA38: ketoreductase
KGD: *α*-ketoglutarate dehydrogenase
LEU: 3-isopropylmalate dehydratase
LPAT: lysophosphatidate acyl-transferase
LPD1: dihydrolipoyl dehydrogenase
LRO1: phospholipid-dependent diacylglycerol acyl-transferases
LSC: succinate-CoA ligase
LYS1: saccharopine dehydrogenase
LYS2: l-aminoadipate-semialdehyde dehydrogenase
LYS9: saccharopine dehydrogenase
LYS12: homoisocitrate dehydrogenase
LYS21: homocitrate synthase
MCT: malonyl-CoA:ACP transacetylase
MDH: malate dehydrogenase
MET17: bifunctional cysteine synthase/*O*-acetylhomoserine aminocarboxypropyltransferase
NQM1, TAL1: sedoheptulose-7-phosphate:d-glyceraldehyde-3-phosphate transaldolase
OAR1: 3-oxoacyl-ACP reductase
OLE1: stearoyl-CoA 9-desaturase
OPI3: ethylene-fatty-acyl-phospholipid synthase
PA: phosphatidate
PAH1: phosphatidate phosphatase
PDA1, PDB1: pyruvate dehydrogenase
PHS1: enoyl-CoA hydratase
PGS1: CDP-diacylglycerol–glycerol-3-phosphate 3-phosphatidyltransferase
PIS1: CDP-diacylglycerol–inositol 3-phosphatidyltransferase
PSD1: phosphatidylserine decarboxylase
PGK1: phosphoglycerate kinase
SCT1, GPT2: glycerol-3-phosphate acyl-transferase
SLC1, ALE1: lyso-phospholipid acyl-transferase
SDH: succinate dehydrogenase
SOL4: 6-phosphogluconolactonase
TDH2: glyceraldehyde-3-phosphate dehydrogenase
TGL: triacylglycerol lipase
TKL1: transketolase
TYR1: prephenate dehydrogenase
ZWF1: glucose-6-phosphate dehydrogenase
