# Supplementary figures and images for: Oleaginicity of the yeast strain Saccharomyces cerevisiae D5A
Source: Biotechnol Biofuels. 2018 Sep 24;11:258. doi: 10.1186/s13068-018-1256-z (PMC6151946; doi:10.1186/s13068-018-1256-z)

## Slide 1
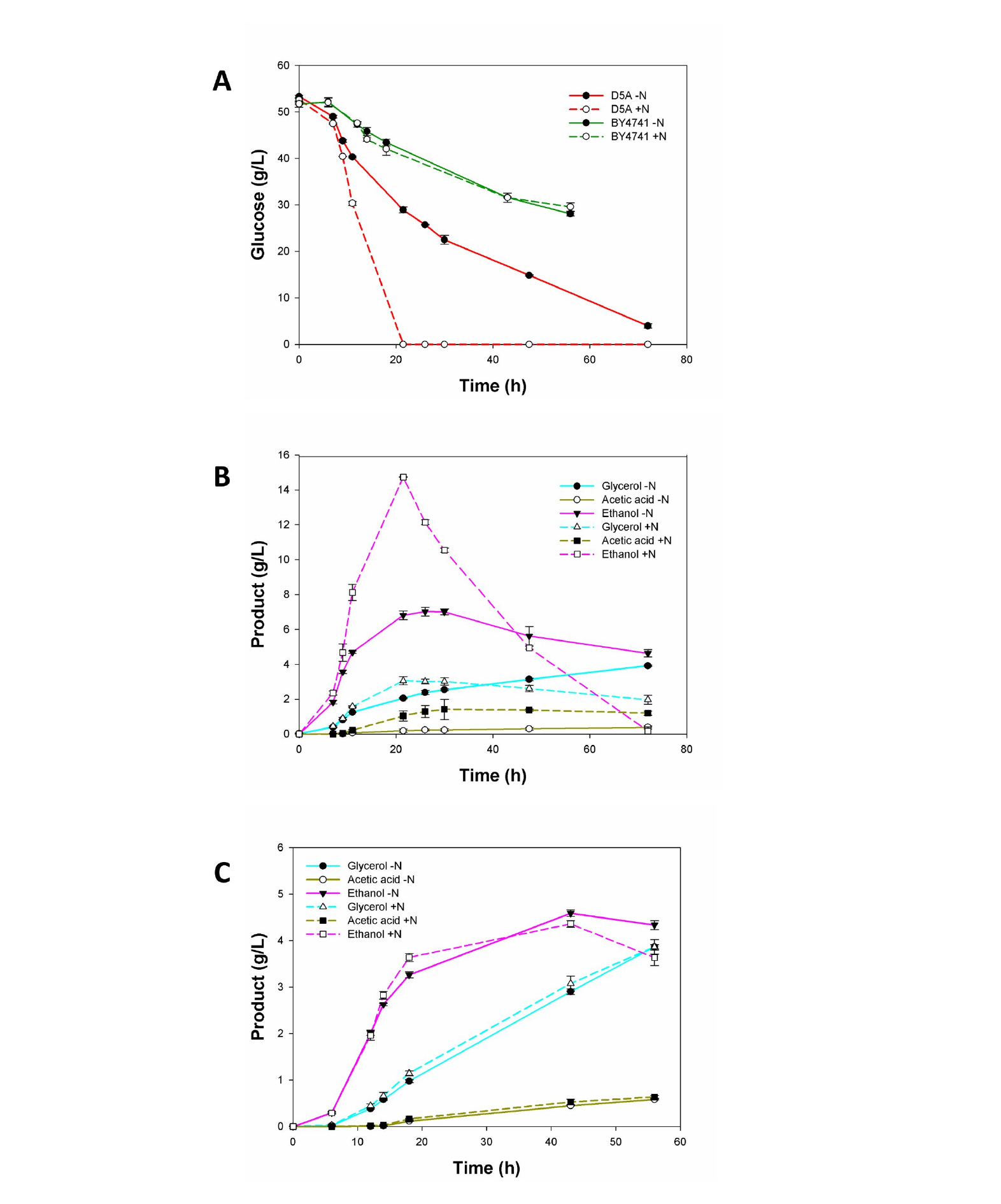

Supplement: Supplementary file 1 — Additional file 1: Figure S1. Glucose consumption in two strains under different nitrogen concentrations (A) and metabolite production in D5A (B) and in BY4741 (C). Data shown as the mean ± standard deviation of duplicate samples. [file 13068_2018_1256_MOESM1_ESM.pptx]
